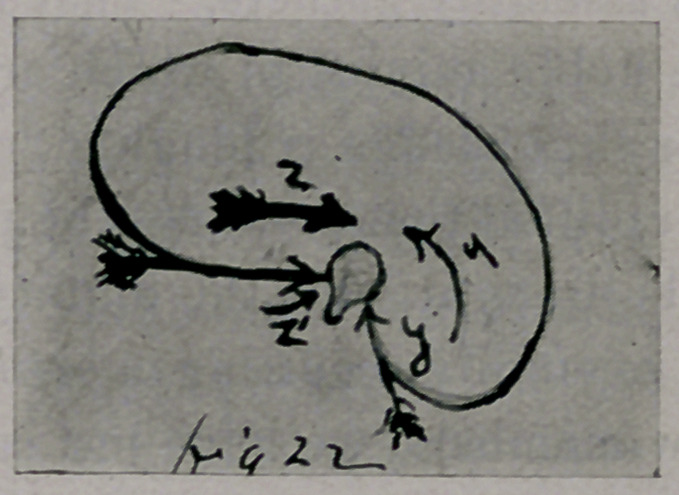# Study of a Case of Lateral Curvature of the Spine. A Report on an Operation for the Deformity

**Published:** 1904-07

**Authors:** Michael Hoke

**Affiliations:** Atlanta, Ga.


ATLANTA

### Journal-Record of Medicine.

**Successor to Atlanta Medical and Surgical Journal, Established 1855, and Southern Medical Record, Established 1870.**

VOL. VI. JULY, 1904. No. 4.

BERNARD WOLFF, M.D., EDITOR, Nos. 319-20 Prudential.

Published Monthly.

M. B. HUTCHINS, M.D., BUSINESS MANAGER, 1007-1008 Century Bldg.

### *ORIGINAL COMMUNICATIONS.*

### STUDY OF A CASE OF LATERAL CURVATURE OF THE SPINE: A REPORT ON AN OPERATION FOR THE DEFORMITY.*

FROM THE ORTHOPEDIC DEPARTMENT OF THE PRESBYTERIAN HOSPITAL.

By MICHAEL HOKE, M.D.,

ATLANTA, GA.

Lateral curvature of the spine without bone deformity is a simple problem. Lateral curvature with osseous deformity in children, whose bones are very flexible, though a more difficult problem, permits correction to a greater or less extent by properly fitting plaster jackets. Osseous deformity in children, adolescents, and adults, whose bones are stiffer and less yielding, has permitted very little, if any, correction. The method of treatment has been about the same as for the younger flexible cases—plaster jackets, remov-

*Presented by invitation at the meeting of the American Orthopedic Association Washington D. C., May 11-14, 1903.

able jackets, exercises, and various combinations of these. The figure of most of the severest cases is as bad when wearing apparatus as when unsupported.

Like all orthopedic surgeons, the writer has been harassed by the uncertainty of prognosis,—*the knowledge that, save in the very young cases, the osseous deformity would not be affected at all by exercises and jackets.*

FIG. 1.

Fourteen months ago the study of this case was begun—an effort to find some operative means by which the deformity of the ribs could be changed, the hump decreased, the depression in the back filled in.

The facts recorded here did not come in the sequence with which they are connected in this paper. Many experiments were made which proved to be valueless. Many designs of instruments and apparatus other than those shown in the illustrations were made, with no result save to indicate how better ones should be made.

FIG. 2.

In this case a change in the bones has been made. A result, though far from perfect, has been obtained, which was not possible without the operative procedure herein reported. The writer is not yet through with the case; but he feels that, with due regard for that conservatism which should always control one's judgment, he may report the work done in connection with this case.

*Patient.*—Age, sixteen years and six months.

*Complaint.*—Curvature of the spine.

*Past History.*—Measles at ten ; mumps at thirteen ; whooping-cough at six ; malaria every spring from nine to thirteen ; scarlet fever at eight—a very mild attack ; "gastritis" at thirteen—attack lasted two months ; has always been delicate.

*Present History.*—May 1, 1902. History of deformity: When she was eight years old, the dressmaker first noticed that one hip and one side were larger than the other. Nothing was done to correct the condition until she was nine years old, when a physician was consulted, under whose care she remained until she was twelve. During these three years she was frail. Exercises were prescribed, which were taken mainly with dumb-bells, pulleys, and other machines. The posture habit received no attention. Her general condition received no medicinal treatment. Under this treatment she thinks the condition remained the same. During this period the deformity was not very noticeable when the clothes were taken off. Observed with the clothes on, the condition was not noticeable. She then went home, and continued the exercises one year. During this period she got worse. She was then taken to an "Institute." A brace was applied, exactly like the one shown in Fig. 1. She went home and came back again at the end of six months to have the pads on the brace changed. During these months she got very much worse. She then returned to the "Institute" to be treated. She remained there one year. The back was steamed, electricity, "two kinds," was given every day, exercises were taken chiefly with machines. She got worse during this time.

*Examination :*—

(a) *General Condition.*

Patient is pale, nervous, tongue coated, constipated, tired all the time, has headaches frequently, sleeps badly.

Heart sounds normal, areas of dullness normal, apex within nipple line, pulse 92 to minute, lungs normal, hemoglobin 65 per cent., no count of corpuscles. Abdomen negative. Urine negative.

(b) *Posture and Curve and Conformation of Body.*

See Fig. 2, kodak picture taken May 16, 1902.

The patient stands with her head inclined toward the right. The right shoulder with pendant arm goes forward: the inferior angle of the right scapula is very prominent, projecting backward about two inches further than the left. The left shoulder with the arm is backward towards the spine: the inferior angle of the scapula is not so prominent as it should be, and lies close to the spine. The left hip is very prominent and forward: it is higher than the right, which is back of its normal position. The weight of the body is thrown on the right leg. When she stands with feet together, the left leg appears shorter than the right. The left heel does not touch the ground. Measurements of the two legs show no real shortening. The apparent shortening is due to tilting of the pelvis and adduction. The outne of the left side shows a deep con avity ; that of the right side is slightly convexed ; the left nipple is a little lower than the right. Over the splenic area the anterior part of the left ribs forms a decided prominence. The left ribs from the fourth to the twelfth are displaced (rotated) forward, so that the area of the thoracic wall in the back formed by these ribs is deeply sunken. This is produced by the fact that the left ribs are rotated for ward, and their backward-arching from the angle out has been flattened. This is shown in Fig. 3, in which the patient was photographed as she was bending forward. The deepest portion of the sunken area lies (presuming

FIG. 3.

the patient to be standing) in front of and below the inferior angle of the scapula. (See crease in skin in Fig. 1, photograph with brace on.) The fifth and sixth ribs are displaced (rotated) further forward and flattened more than the others involved on the left side of the spine.

The ribs on the right side from the fourth to the twelfth form a prominence. This decided deformity of the right ribs is seen in Fig. 3. The prominence is produced by rotation backwards of the ribs and by the fact that the normal-postero-convex curve of the ribs near the angle has been greatly increased.

The curve of the spine is an S-shaped one, extending throughout the length of the spine.

Beginning at the base of the skull, with the head inclined to the right, the spine curves to the left to the first dorsal, then slightly to the right to the fourth dorsal, then very acutely to the right, as if, with the articulation of the fourth and fifth dorsal vertebræ as the plane of motion, the section of the body from the fourth dorsal vertebræ (inclusive) up had been shifted to the left upon the lower segment (from fifth dorsal vertebra, inclusive, down). Thus the fifth spine is about one inch below and to the right of the fourth spine. Then this curve to the right continues to the eighth vertebra, at which point it turns to the left, crossing a plumb at the twelfth, and continues into the lumbar region.

The patient sits with the head, shoulders, arm and hips in the same position relative to one another as they are when standing. The normal lumbar curve has been greatly increased, so that the patient when standing, is very "sway-back." The costal margin in front is for that reason very prominent.

If a perpendicular plane is let fall through the transverse diameters of hips, shoulders and chest of a normal individual, these planes will be approximately parallel. Here they are inclined to one another.

Fig. 4.—Let line H H represent the projection of a plane passed perpendicularly through the transverse diameter of the hips in the position they ought to occupy. Pass similar planes through the transverse diameters of the hips, thorax, and shoulders in their present positions, and project them upon the ground. Let line H H be the basis from which the rotations of the hips, thorax, and shoulders of the patient, can be approximately estimated. Then LH, etc., represent the positions of left hip, left side of thorax, and left shoulder in the postures the patient assumes in sitting and standing, and the arrows *lh*, etc., the directions in which the left hip, etc., have rotated. RS, etc., represent the right shoulders, etc. The torsion in

FIG. 5.

the body can be appreciated from this diagram, which is only an eye estimate of the rotation of the parts mentioned.

There is no rotation forward, and consequent flattening of the left first, second, third, and fourth ribs, and no bending backward of the first, second, and third ribs on the right side. The curve backward in the fourth rib on the right is a little increased, but not enough to be striking. The section of the chest above the fifth rib may be regarded as structurally symmetrical, though it shows a postural curve. Below this segment the symmetry of the skeleton has been produced.

The greatest change in the spine is at the fifth, sixth, seventh and eighth vertebræ. These vertebræ are rotated more to the right than the others. The deepest part of the sunken area in the left side arises from the fact that the fifth, sixth, seventh and eighth ribs are displaced farther forward and flattened more than the other ribs on the left side. The greatest amount of bending backward of the ribs on the right side has occurred in the fifth, sixth, seventh and eighth ribs. The section of the thorax formed by these vertebræ and ribs has become most markedly deformed.

The segment of the chest from the fourth to the eighth vertebræ seems to take no part in the motions of the spine: this section seems fixed. The spine is unnaturally flexible at about the tenth dorsal to the second lumbar vertebra. Lateral flexion of the body is accomplished mainly by the motion between the tenth, eleventh and twelfth dorsal and first lumbar vertebræ. The trunk can be laterally flexed to the right about half as far as the left.

The experiments below were made upon a *cadaver* taken without selection from a number in the head-house. The body was that of a man, who had been a laborer. The ribs were thick, hard and resistant, the ligaments strong.

The muscles of the back were cut away. *The sternum with the ribs attached was left intact.* The thoracic viscera were not removed. The pelvis was made steady in a box.

The bony part of the thoracic cage is made of vertebræ, cartilages, ribs and sternum. Let a "thoracic element" be a vertebra, and its attached ribs not united or united in front by the sternum, or indirectly by attachment to the costal border. The shape of such an element is shown in Fig. 5. Fig. 5 is a photograph of the fifth element taken from the subject after the experiments below were made. Mark the centre of the vertebra, mark the opposite point on the sternal portion of the element. Find points on the ribs equally distant from the sternal point. These points are equally distant from the centre of the vetebra.

Fig. 5. If *bd* and *be* are equal, then *ad* and *ae* will be equal. The symmetry of the figure is striking. Support the element upon a rod in the position of its antero-posterior diameter. The element balances long enough to be photographed. (See Fig. 6.) These two experiments were made with the first, second, third, fourth and fifth elements. The results were analogous in each instance. The first seven elements are to be called "closed elements" (complete bony rings) for the reason that the ribs of each are united to their fellows by the sternum. These elements are rigid, and they are rigidly held together in the front by the sternum. The firmness of the thoracic cage behind, along the spine, must depend upon the rigidity of each element, and the firmness with which the neighboring vertebræ and ribs, out as far as the angles, are held together by the various ligaments.

FIG. 6.

The eighth, ninth, and tenth elements are indirectly "closed," since they are joined to the continuous costal border; but these elements are of necessity not as rigid as the first seven. The eleventh and twelfth are not "closed."

FIG. 7.

Fig. 7 shows the subject with straight spine and symmetrical thorax sufficiently suspended to keep it from bending forward.

Fig. 8. The thumb of the left hand is pressing forward upon the end of the left twelfth rib. The end of the left twelfth rib could be pressed forward easily some little distance. The index finger of the right hand points to the right twelfth rib, which rotated backward. Pressure forward was made upon the left eleventh and upon the tenth ribs at the angle in the same manner. There was much less discursion forward of the left tenth rib than the left eleventh and twelfth, less rotation backward of the right tenth rib than of the right eleventh and twelfth ribs when subjected to analogous pressure conditions. Pressure forward upon the left ninth, eighth, seventh, and sixth ribs, gave progressively less discursion forward of the ribs pressed upon than was seen when the foregoing ribs were similarly pressed forward, and progressively less rotation backward of the corresponding right ribs. In the eighth, seventh, and sixth ribs the movement produced was very slight. Extremely little rotation of the vertebræ was produced.

Fig. 9. The eighth rib was completely severed at the point marked by the thumb in the illustration. Forward pressure was made by the thumb

FIG. 8.

FIG. 9.

FIG. 10.

FIG. 11.

upon the end nearest to and still attached to the spine. This end could be pressed forward three-quarters of an inch or more easily. There was a little deflection of the eighth vertebral spine to the left, indicating rotation of the vertebral body to the right, perceptible to the eye, but not enough to show in the photograph. There was also much more rotation backward of the eighth and ninth ribs than was seen when pressure was made at the same site before the rib was cut.

The index finger is making pressure at the same site as the thumb in Fig. 10. The second finger points to a discursion forward of the rib below (pulled forward by the anterior corto-transverse ligament between it and the vertebra above). This was perceptible to the eye. The movement was, however, not sufficient to show in the photograph.

Fig. 11. The left seventh, eighth, ninth and tenth ribs were cut where the rib turns forward. Pressure was made by the fist upon the ends nearest the spine. These ends could be easily pressed forward an inch. There was enough rotation of the vertebræ to the right to be shown in the photograph (see deflection of spine of the vertebræ to the left). There was quite a good deal of bulging backwards of the corresponding ribs on the right side. The rotation of the ribs backward on the right was much greater relatively than

FIG. 12.

the discursion forward of the fragments pressed upon by the fist. The corresponding clinical observation (the relatively greater hump on one side than depression on the other) can be made by the examination of any osseously deformed case. It was far easier to bend the spine laterally than it was before the ribs were cut.

Fig. 12. A knife was passed between the transverse processes on each side of the vertebral column towards the spine, cutting the anterior corto-transverse ligaments. The weight of the thorax easily curved the spine laterally, and rotation of the elements was vastly easier. The eleventh and twelfth elements are not closed figures.

*Deductions.*

Pressure forward at *a* through the mediation of the middle costo-transverse ligament and vertebra rotates the vertebra to the right and the opposite rib backward. A slight rotation of the vertebra is sufficient to produce a greater arc of motion at *b*, since *b* is the end of the long arm of the lever. A closed element, since it is a bony ring, can not give in to pressure at a point without yielding somewhere else in the ring. The ribs in the closed

FIG. 13.

elements are to be regarded as props to the spine. They are forces, since they transmit the weight of the thorax, its contents, the head and uppe extremities, to the spine. In the normal, symmetrical, closed element the force of corresponding right and left ribs is applied to the spine in the same plane. (See Fig. 14, arrows *a* and *b*.) They must balance one another.

When forward pressure was made near the left angle of the rib of a complete closed element, the ribs of which were thick and resistant, there was extremely little yielding at the point of pressure, and extremely little rotation of the attached vertebra, and extremely little rotation backward of the opposite rib, because the element was rigid, it would not yield to pressure. When, however, the left rib of a closed element was cut and forward pressure was made on the fragment nearest the spine, that fragment could be easily pushed forward three-quarters of an inch or more, the attached vertebra could be rotated to the right, and the corresponding

FIG. 14.

FIG. 15.

right rib rotated backwards. In other words, pressure at *a*, Fig. 15, produced rotation of the vertebra in direction of arrow *b* and greater rotation backward of right rib in direction of *c*. Here, again, the greater discussion at *c* appears, as the result of this point being at the end of the long arm of the lever. Thus, if the ribs are firm, pressure forward on the angle of a rib can but little affect the figure of the element by producing yielding at the point of pressure and rotation of the vertebra. But, when the equilibrium of the forces on either side of the vertebra (from integrity of the ribs) is destroyed by cutting a rib, then it is possible to rotate the vertebra to produce backward motion of the angle of the rib opposite to the one cut. Pressure at *c* produced rotation the reverse of that produced by pressure at *a*. The same result was obtained, as mentioned above, but to a greater degree, progressively, as more ribs were cut, the ligaments being still undisturbed. Thus, so long as each element is symmetrical and unyielding, the ribs possess their incremental power of preventing lateral curvature, rotation of the spine, and deformity of the thoracic cage.

FIG. 16.

But, if an element yields, the ribs being flexible or having been made so artificially, then by pressure at the right place one may influence the element by corrective force. Each rib in a symmetrical element must equally oppose the force of its fellow on the opposite side, the two ribs equally bracing the vertebra.

When the anterior costo-transverse ligaments connecting a number of elements were severed with the knife, it was very easy to bend the spine laterally, and to rotate the whole thoracic cage to an extent limited, but greater than normal.

Let *abc* be a rod resting at its centre *b* upon a pivot; *d* and *e* are equal weights suspended from the ends; *xy*, a pedestal; *op* and *o′p′*, slightly elastic bands of equal size. Depress *c*, remove the pressure: as a result, the rod will oscillate and finally come to rest. Add to the weight *e*, then *c* will descend in some proportion to the weight added, and the yielding of elastic band *op*. Remove additional weight, the rod will oscillate and come to rest again. Turn back, and look at Fig. 7, a symmetrical element balancing on a rod. All symmetrical elements are balanced, and a symmetrical thorax is balanced upon the spine.

Observe the analogy of Fig. 17 with Fig. 16: *x* equals pedestal and pivot, a vertebra of an element; element *abc* and elements above and head and extremities above pedestal and pivot are comparable to rod *abc* and weights. A similar relationship exists between all elements. Then the anterior costo-transverse ligaments and the muscles of the back and side of trunk correspond to the elastic bands *op* and *o′p′*. Think how a bad posture may affect this figure, adding more weight to the right or left side of the spine. The other ligaments of the spine were left intact in the above experiment (producing lateral bending so easily after the anterior costo-transverse ligaments were cut), and likewise the fascia between the ribs from the angle of the ribs to the sternum. These must likewise play their incremental part in holding the symmetrical elements together. It did not seem worth while—likely it could not be done--to try to determine exactly what part each one of these ligaments played in holding the frame upright. All play their part, but it seemed that among the ligaments the anterior costo-transverse must play a very prominent part in preventing lateral bending and rotation beyond a certain limit, regarded by the eye as normal; and how far the rotations occurred, except "much" or "little," seemed immaterial, too,—a detail that would vary with every *cadaver* and every living subject, dependent (presuming them to be symmetrical) upon the thickness and consequent rigidity of the subject's ribs and upon the strength, length and elasticity of the anterior costo-transverse ligaments mainly, and other ligaments of the spine and attached ribs, and the fascia between the ribs.

FIG. 17.

Think how easy it is to bend the neck in any direction, and to rotate it: there are no ribs there. Think of the mobility of the lumbo-dorsal junction: there are no closed elements there. Notice the fact that between the shoulder-blades there is less mobility than in any other part of the spine. In this thoracic section are found the most rigidly closed elements, joined in front by the sternum. The component ribs are thicker, wider, stronger and less yielding than their fellows in the thoracic wall. Observe the skeleton of a symmetrical subject. (See Fig. 18.) The symmetry of the bony cage thorax is really marvelous. Take any point on a rib. Find analogous point on its opposite fellow. These two points are equally distant from the spine; and, if not in the same horizontal plane, they are very near it. Corresponding intercostal spaces are of the same width at corresponding points, etc.

The following facts are of great importance: The distance from the head to the angle of the ribs increases from the first to the eighth, and then decreases again. At the angles the ribs turn for-

FIG. 18.

ward: thus the surface of the cage from the first eight angles forward forms a plane inclined forward and outward. These angles lie beneath the vertebral border of the shoulder-blade. Naturally, on a symmetrical subject standing erect the shoulder-blade glides forward to a normal degree over this inclined plane. If the shoulder on the normal thorax should glide backwards, it must ride over an eminence. The two upper extremities form a good portion of the superincumbent weight upon the spine. The posture of the shoulder within certain limits is subject to normal variation. Departures from the normal posture of the shoulders produce a symmetrical distribution of the weight of these extremities upon the spine. It has been demonstrated that weight falling obliquely upon the spinal column can produce a rotary lateral curvature. (Bradford and Lovett, "Orthopedic Surgery," 2d edition, 1899, p. 94.)

FIG. 19.

So long as this patient grew up with no bad postural habit and with fair health, with her ligaments strong enough to keep the thorax balanced, her muscles in good condition, so that they were used co-ordinately, the development of each one of these elements was a symmetrical development, and she was straight. But habitual

2

posture placed the weight of the head, upper extremities and thorax upon the spinal column obliquely. Bad health, anemia kept her constantly tired, her ligaments and muscles weak, her bones soft. She acquired the habit when standing and sitting, when reading or writing, of holding the head to the right, of rotating the left shoulder backward, the right forward, and left hip up and forward, the right backward. As a result of this, the superincumbent weight fell obliquely upon the column in the direction of the arrow in Fig. 19. In this picture the patient is photographed standing. The paper arrow stuck on the chest shows the direction of the force of the superincumbent weight. The posture with reference to the spine was one of flexion forward and lateral flexion to the left. Under this influence, acting through a number of years, the spine bent with the convexity of the curve to the right (in the dorsal region). Thus the bodies of the vertebræ rotated to the right; and the ribs rotated and bent, too, until the parts of each thoracic element under the superincumbent weight were acted upon in the direction of the arrows in Fig. 20.

Arrow *a* represents the action of the slanting force of the body weight upon each thoracic element from the fifth inclusive down. From this vertebræ rotated in the direction of the arrow *b;* the spine, arrow *c.* The right ribs were acted upon by forces (arrows) *a* and *d;* the left ribs, arrows *e* and *b.*

Fig 20

Fig 21

The body weight acted in this manner for several years. Thus each thoracic element was by these forces changed from the symmetrical shape to the deformed in Fig. 21.

In the symmetrical element the two ribs (forces) are attached (applied) to the spine at analogous points on the sides of the vertebræ. Here is the deformed thorax, in these deformed elements, all like Fig. 21, not only were the elements deformed, as shown in Fig. 22, but the direction of the application of the forces (ribs) to the spine was different on the two sides. The point of application of those on the left was anterior to the similar point of application of their fellows on the right; and the forces (ribs) were applied at differently inclined angles, which under the influence of weight must make the chest rotate to the right.

The sum total of the changes in the elements produced the deformity seen in Figs. 2 and 19, standing, and Fig. 3, bending forward. Thus the back to the left of the spine was sunken in from the fourth rib down; to the right of the spine the prominence of the ribs extended from fourth rib down; the chest wall was flattened beneath the right axilla; a prominence was produced over the left costal margin and adjacent part of ribs in front. This combined skeletal deformity and bad posture gave the figure of the patient.

The barbarous brace shown in Fig. 1 held her in this deformed posture, and by vicious pressure upon the thoracic wall increased every feature of the deformity. The photograph illustrates the harm that may be done by such grossly ignorant application of apparatus.

In the beginning the problem was not clear; but, with the hope that it would become clear, the effort for several months—May, June, July, and August—was to render the spine as flexible as possible. Exercises were given daily for three months. These were movements of the lower extremities, symmetrical extension exercises for the back, combined exercises of the legs and abdomen, with the intention of reducing the exaggerated lumbar curve, neck exercises, and arm movements. Suspension was combined with manipulation of the trunk by the hands, in order by counter-rotation (rotation to the left) efforts to stretch the contracted ligaments better.

When the writer, standing behind the patient, grasped her pelvis at the left anterior superior spine with his left hand, and her chest wall over the prominence of the right side with his right hand, and rotated the former backward and the latter forward, a much more corrected attitude could be assumed by the patient—the back flatter, the thorax more directly over the hips, the depression shallower, the rib bump less prominent, and, when the hands were removed, the body slid obliquely to the right, *and twisted to the right.* When these counter-rotation efforts were made at intervals during the time the patient was exercising in the office, it made it easier for her to exercise in the more correct posture. In other words, when trying to stand straight, to flatten the back, to straighten out the curve, to hold the body directly over the pelvis, what opposed her efforts was the *tendency to twist around the spine to the right*—torsion—and in proportion to the degree this was corrected for her by the writer's efforts with his hands was she enabled to assume a better posture; and the reason she could not assume such a degree of improved posture alone was the fact that her own muscles lacked just as much power to rotate the body and spine back towards the normal posture as the writer put in his efforts to assist her. This was sufficient to make him "strain." Some appreciation of the task placed upon her muscles can thus be had. Using all his force, the writer was not able to twist the body much to the left. *There was a great resistanee to this effort.*

The mechanics of this was as follows (it is most important to understand this clearly):

It was seen by examination that the width of corresponding intercostal spaces is the same in a normal thorax (fascia and intercostal muscles fill this). The same is true of the space between corresponding transverse processes, which means that the anterior costo-transverse ligaments between corresponding transverse processes and ribs are of equal length, balancing, behind, the anchorage of one element to another. Under the obliquely acting superincumbent weight each element from the fifth inclusive down had been deformed as above, the ribs on the left were crowded close together, narrowing the intercostal spaces (shortening the fascia and muscles), narrowing the left inter-transverse spaces (shortening the anterior costo-transverse ligaments of the left side), and the tendinous attachments of the deep muscles of the back extending across these spaces from the vertebral spine out to the angle. The same structures on the right side of the spine were stretched.

All the exercises could possibly accomplish was to stretch the ligaments, etc., on the left side, and to develop the muscles of the back. The counter-rotation efforts with the writer's hands helped to further stretch all the ligaments, and made it easier for her muscles to hold her straighter. The ligaments having been stretched, giving greater latitude of motion of the parts towards the normal, and the muscles having been toned up, a certain amount of rotation and lateral flexion could be corrected by her muscles. But the bone deformity of each element had not been changed in the least. The left rib of each element was still sunken into the left of its vertebra as much as formerly. It has been shown that the ribs are props to the spine, that each rib is a force acting on the spine, that in a normal thorax, each element being symmetrical and these ribs being of the same size and applied to the spine in the same plane, they have no advantage over one another. Here,

Fig 22

however, the situation was different. As seen in Fig. 22, the line of action of the left ribs (force applied to spine) were now all anterior to the line of action of the right ribs. In addition the left ribs were almost straight. Thus all the deformed left ribs made a "straight thrust" against the spine, the right ribs an oblique thrust.

In attempting to counter-rotate each element further, to correct the torsion, to change the shape of each element, the effort to rotate the spine in direction of *y* met rib *z*, greater than *y*; for *y* was limited to the extent the patient could stand pressure and pain. (See Fig. 22.)

The rib being unyielding, the result desired could not be accomplished until *z* was weakened. This could not be done until *z* was severed somewhere, probably close to the spine. Upon this principle depended the only possible hope.

(TO BE CONTINUED.)

## Figures and Tables

**Fig. 1. f1:**
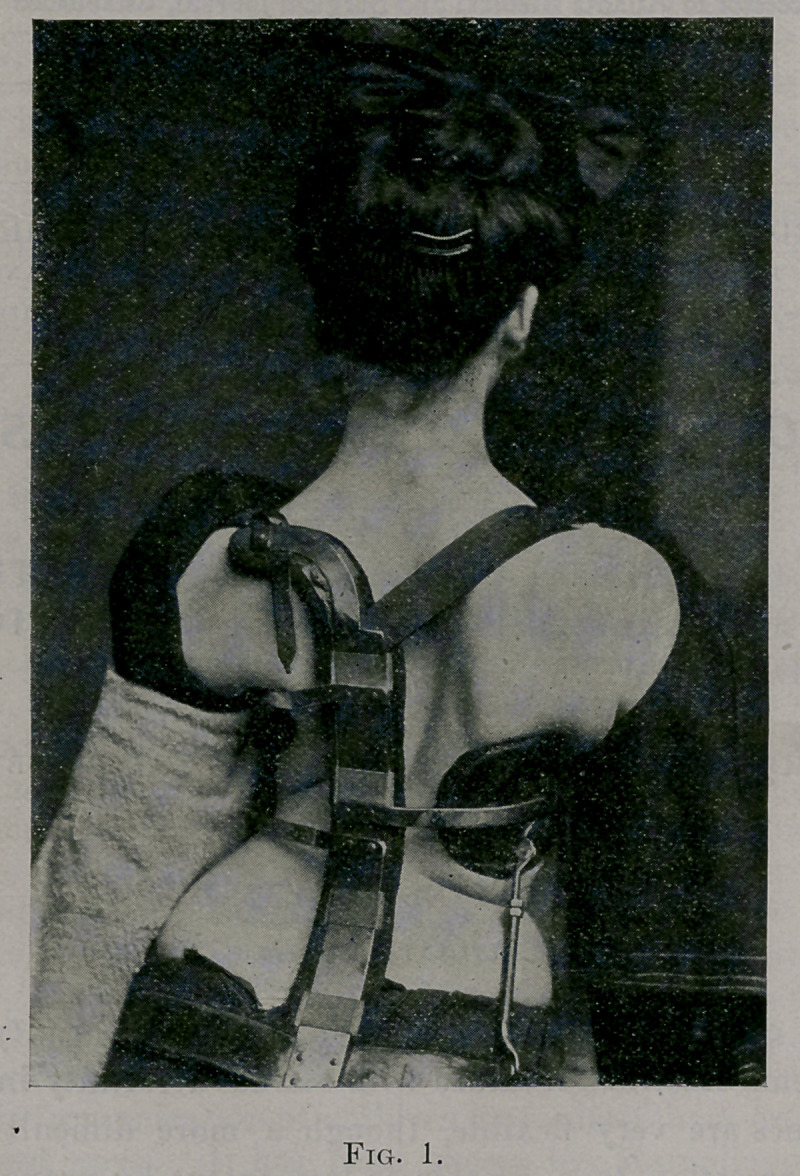


**Fig. 2. f2:**
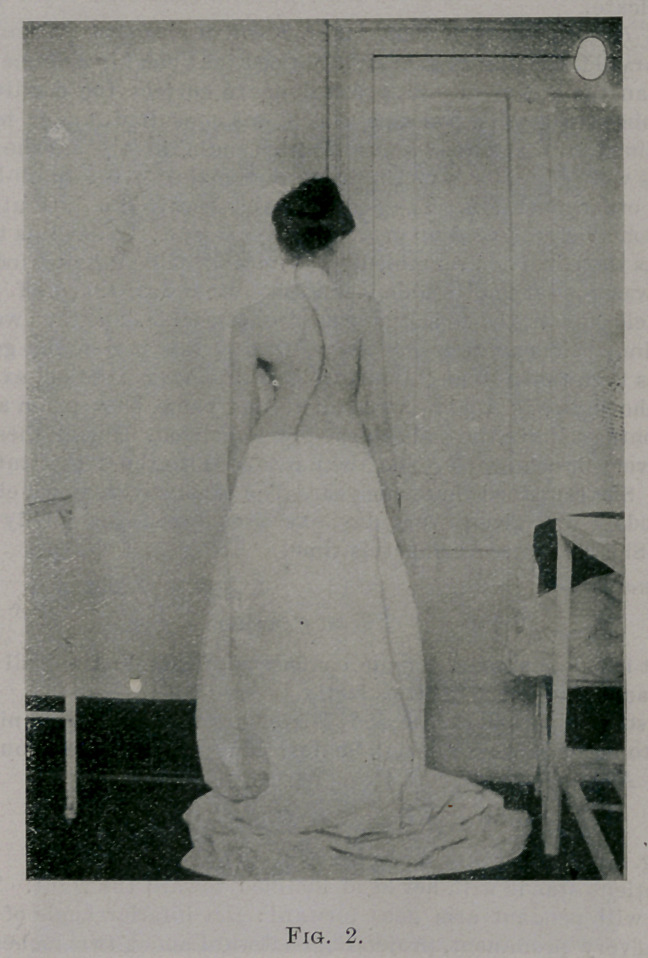


**Fig. 3. f3:**
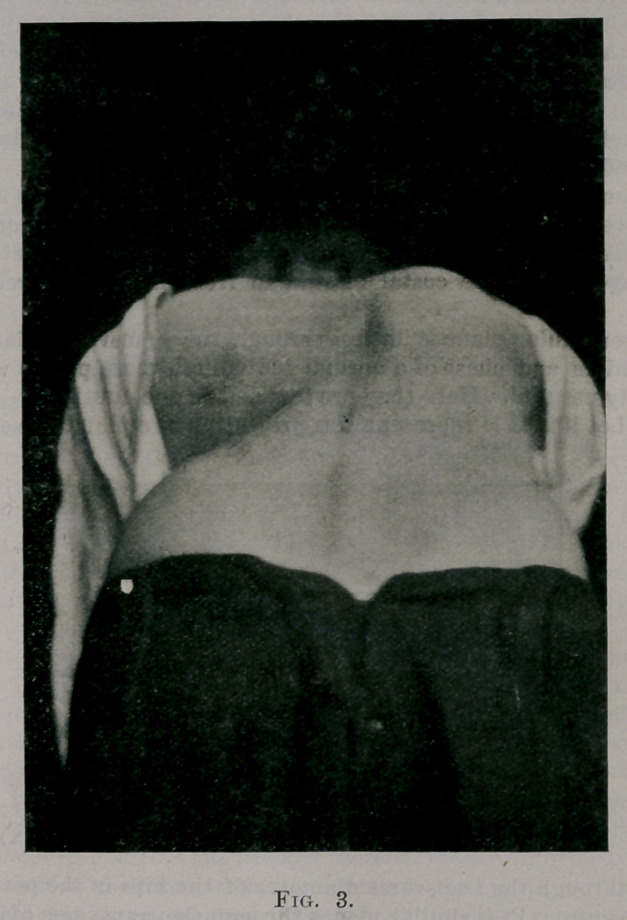


**Fig. 4. f4:**
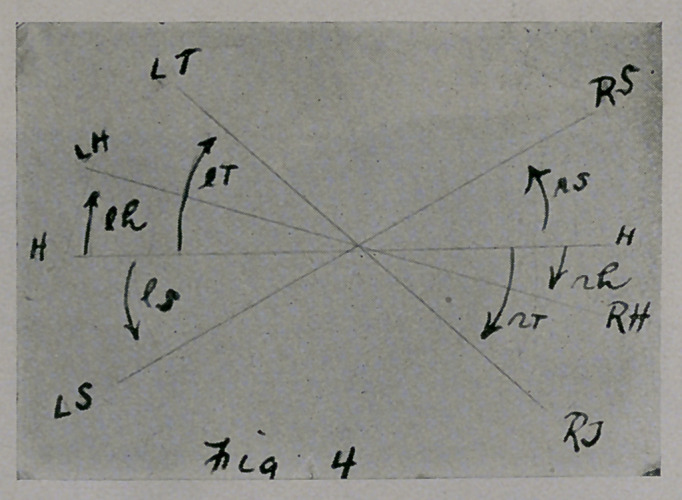


**Fig. 5. f5:**
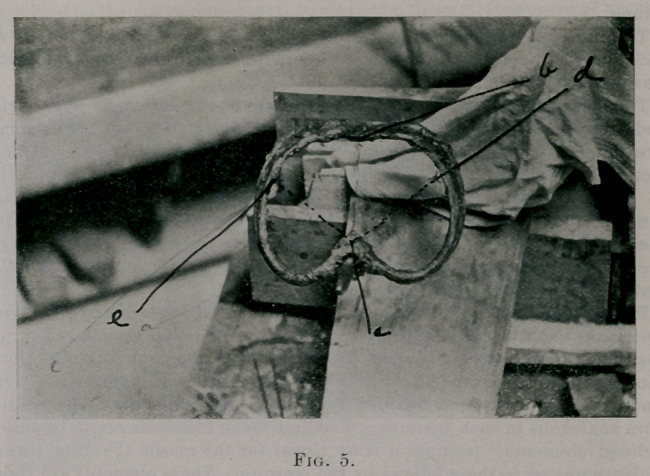


**Fig. 6. f6:**
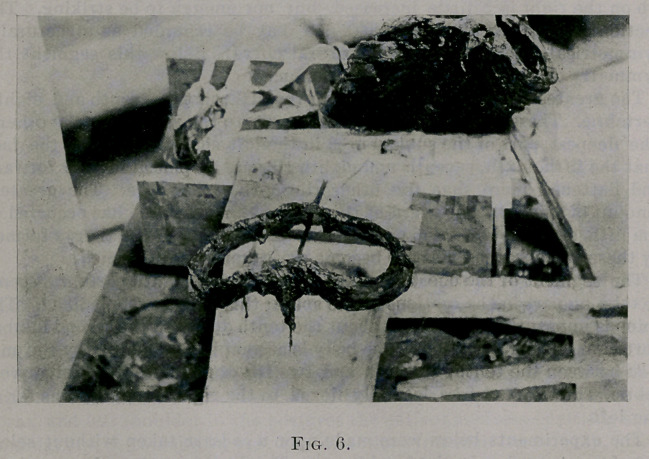


**Fig. 7. f7:**
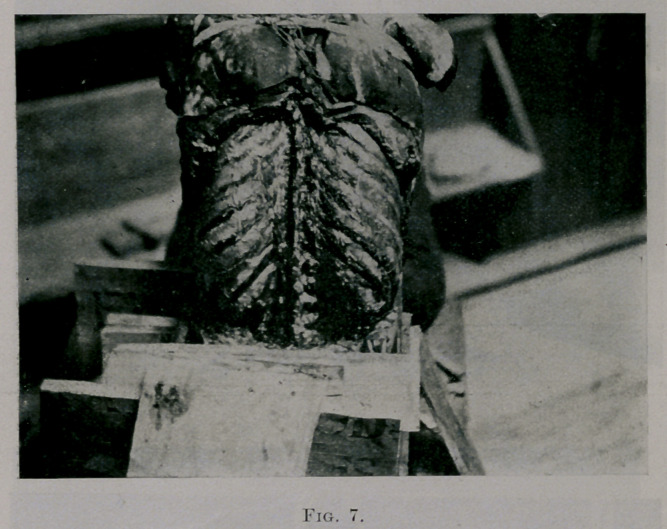


**Fig. 8. f8:**
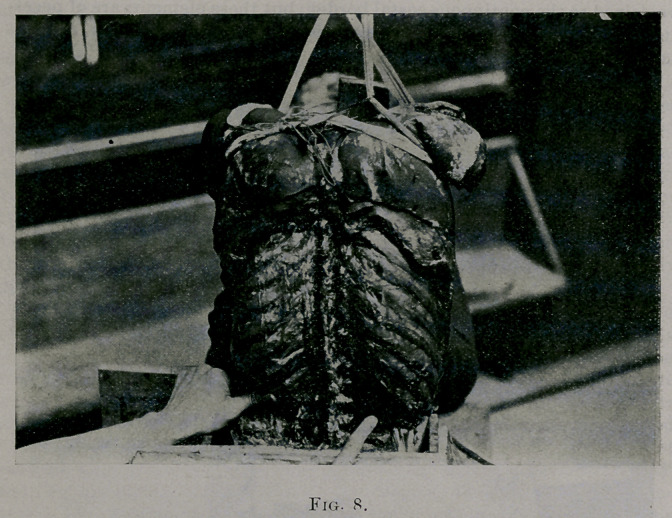


**Fig. 9. f9:**
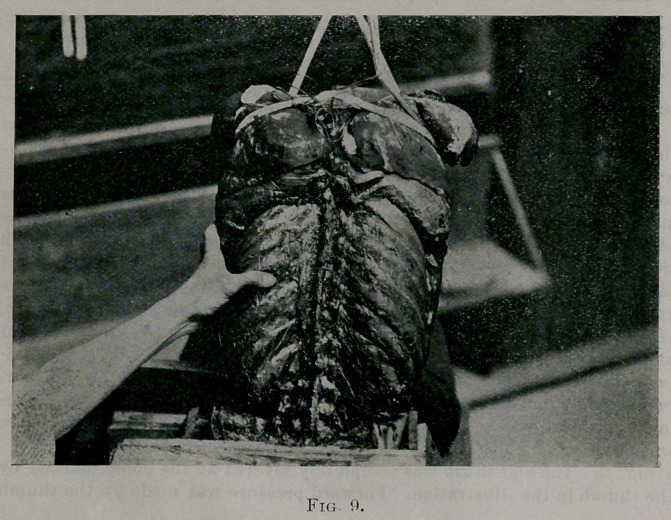


**Fig. 10. f10:**
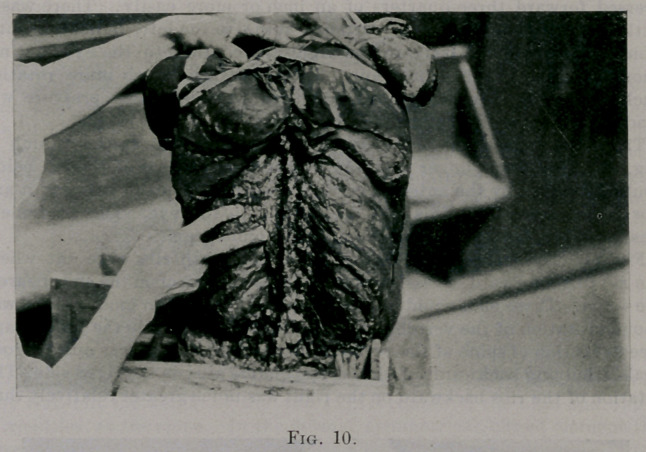


**Fig. 11. f11:**
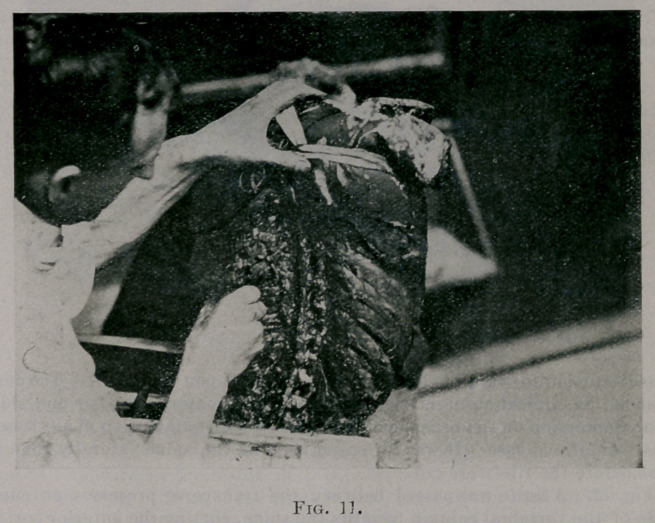


**Fig. 12. f12:**
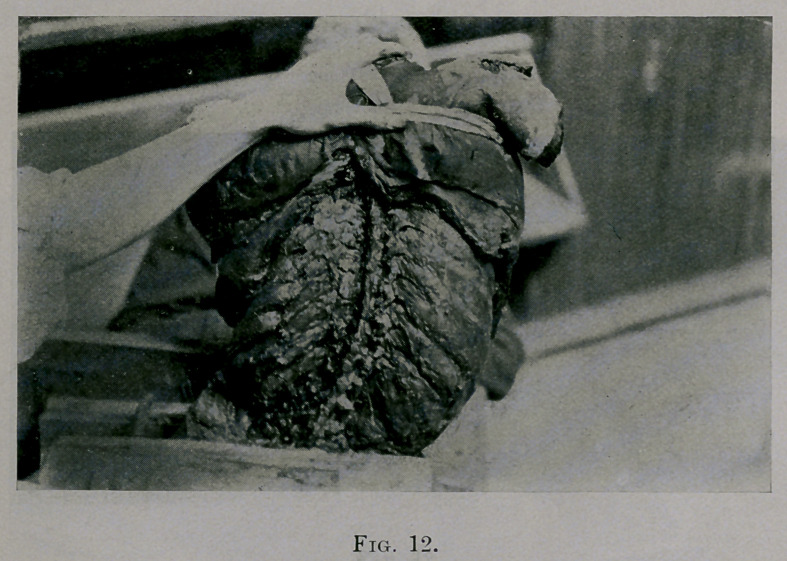


**Fig. 13. f13:**
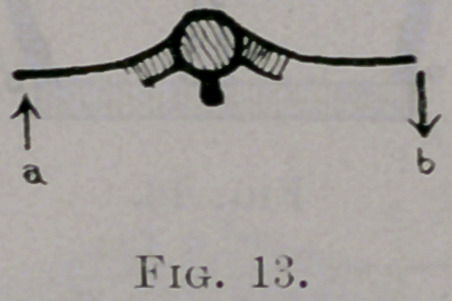


**Fig. 14. f14:**
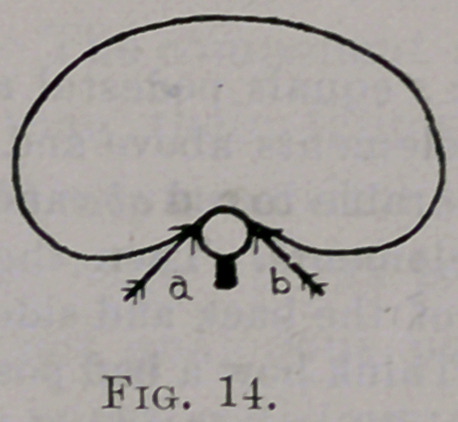


**Fig. 15. f15:**
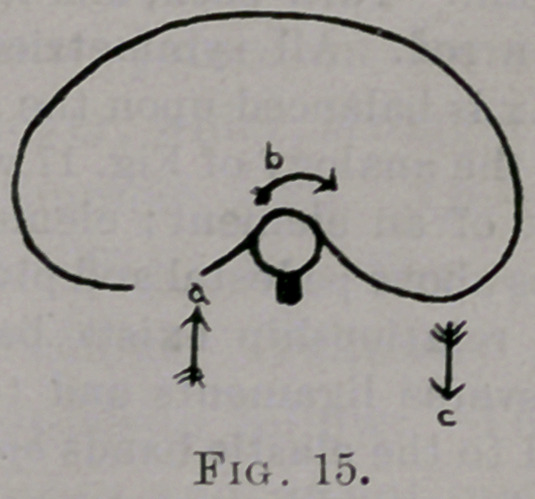


**Fig. 16. f16:**
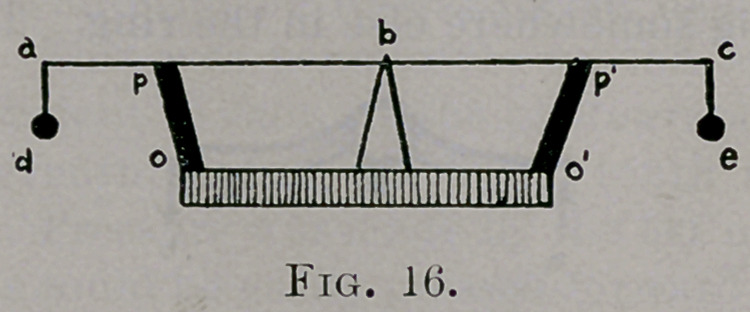


**Fig. 17. f17:**
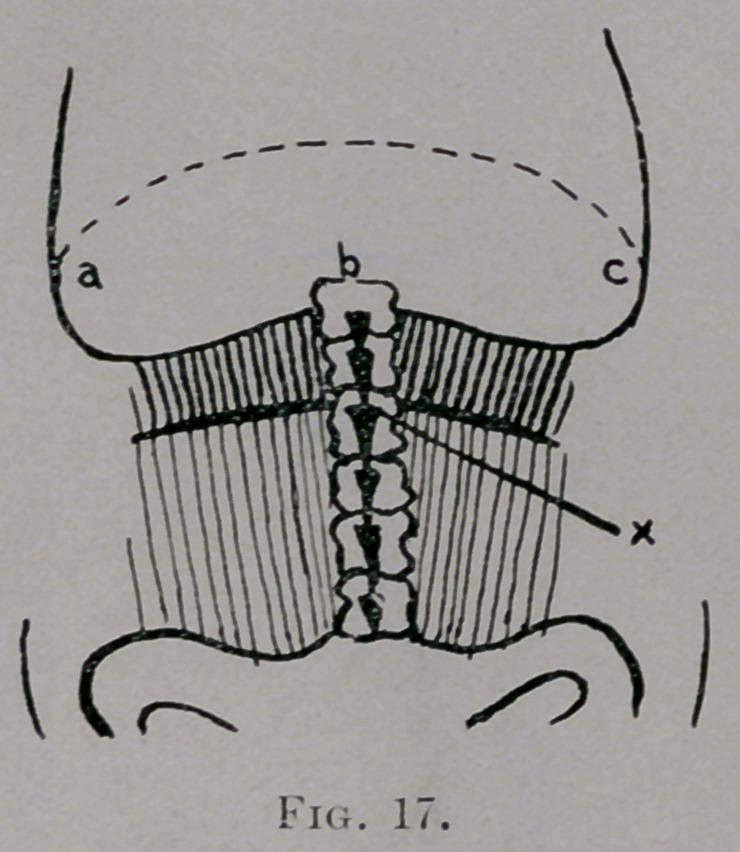


**Fig. 18. f18:**
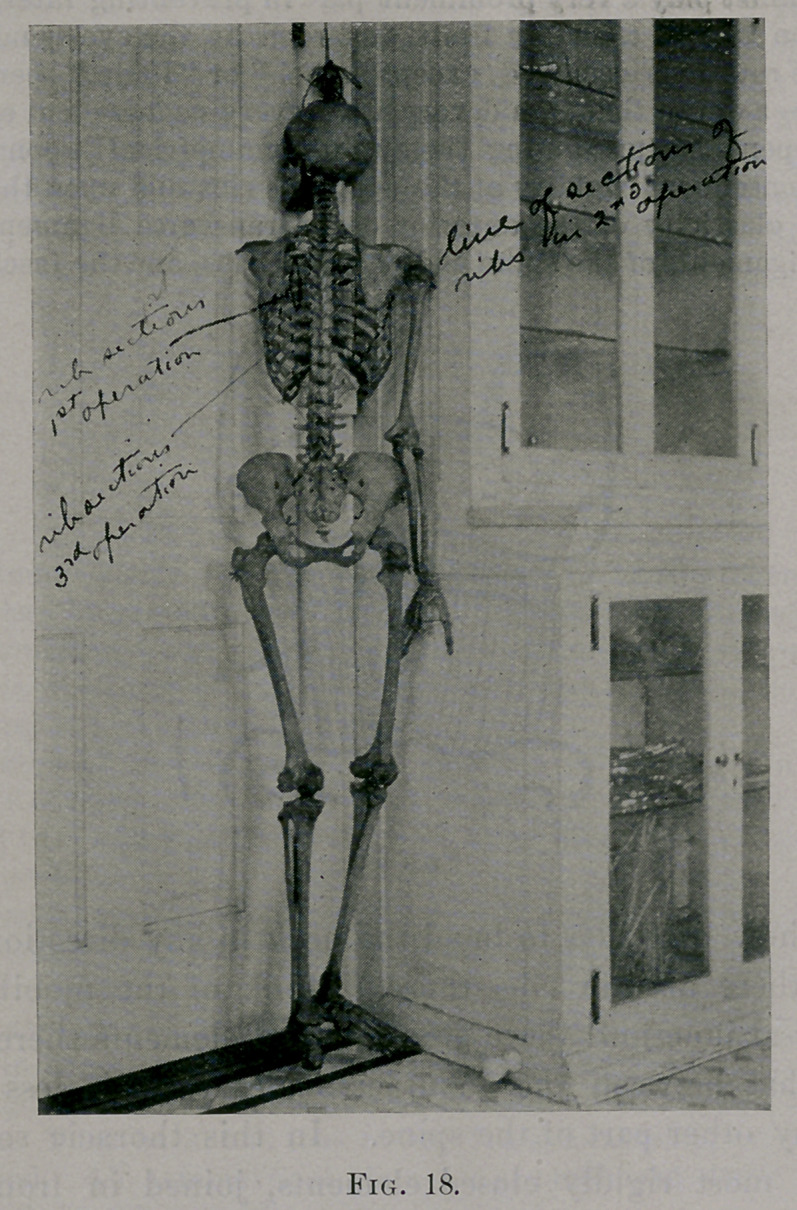


**Fig. 19. f19:**
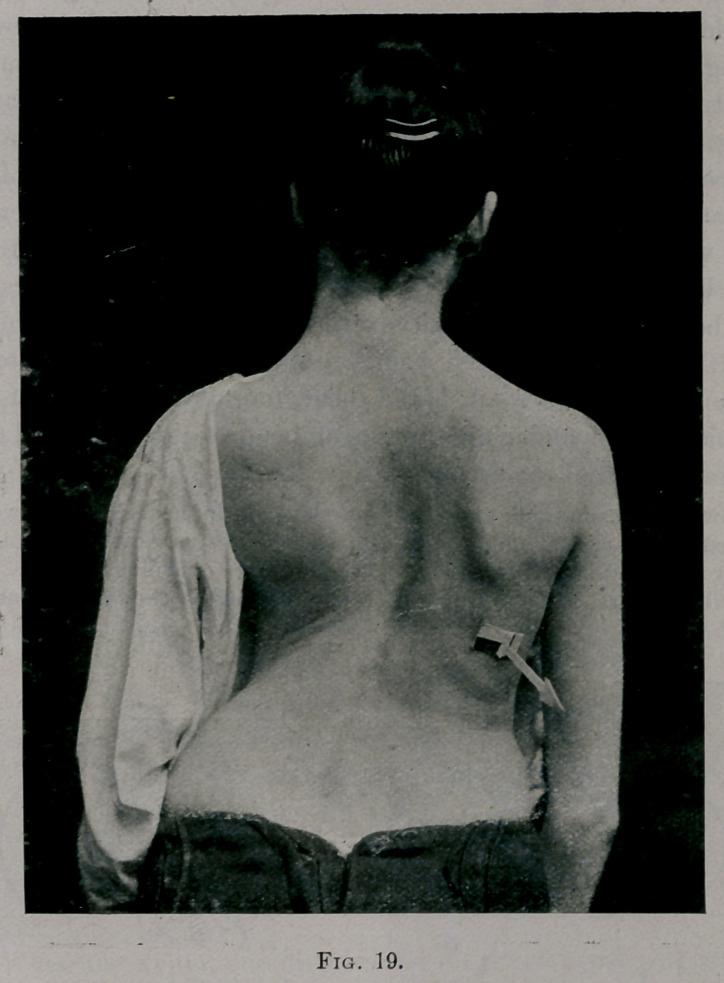


**Fig. 20. f20:**
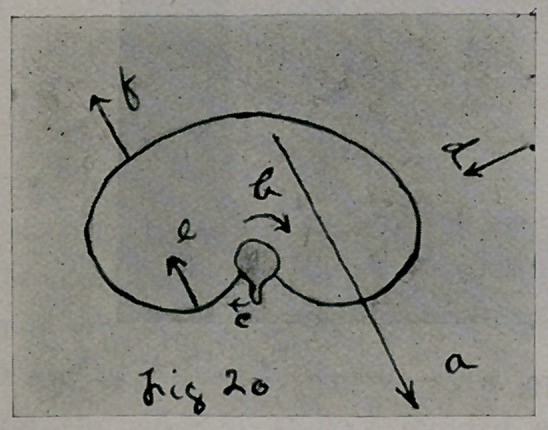


**Fig. 21. f21:**
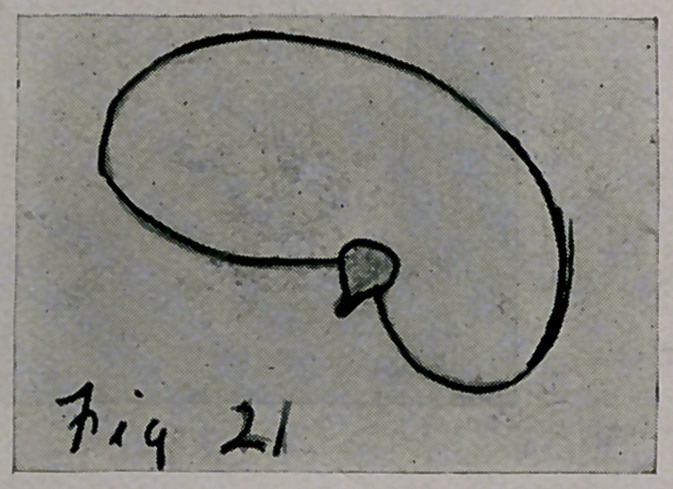


**Fig. 22. f22:**